# Incidence and predictors of mortality among patients admitted to adult intensive care unit at public hospitals in Western Ethiopia: a retrospective cohort study

**DOI:** 10.3389/fmed.2024.1370729

**Published:** 2024-11-20

**Authors:** Fetene Kebede, Getu Mosisa, Mekdes Yilma

**Affiliations:** ^1^Disease Prevention and Control Department, Jima Arjo District Health Office, Oromia, Ethiopia; ^2^Department of Nursing, Institute of Health Sciences, Wollega University, Nekemte, Ethiopia; ^3^Department of Public Health, Institute of Health Sciences, Wollega University, Nekemte, Ethiopia

**Keywords:** ICU, mortality, incidence, predictors, Western Ethiopia

## Abstract

**Introduction:**

In resource-constrained countries, the incidence of mortality among patients admitted to adult intensive care units is higher than that in developed countries, which has a physical, economic, and emotional impact on the lives of patients and their families. However, there is limited evidence on factors related to nursing care that can potentially contribute to predicting and reducing mortality rates in intensive care units. Therefore, this study aimed to assess the incidence of mortality and its predictors in patients admitted to an adult intensive care unit.

**Methods:**

A retrospective cohort study was conducted among 403 patients admitted to the adult intensive care unit from 1 January 2021 to 31 December 2021. The collected data were entered into Epi Data Manager v4.6.0.6 and exported to SPSS version 24 for analysis. Binary logistic regression was used to identify the predictors of mortality. Variables with a *p*-value less than 0.25 in bivariable logistic regression were selected for multivariable logistic regression. A *p*-value <0.05 was used to indicate a significant association in multivariable analysis. Finally, the adjusted relative risk (RR) with 95% CI was calculated.

**Results:**

A total of 403 patients were included in the analysis. The cumulative incidence of death was 40.9% (95% CI 36, 45.9%). Mortality was significantly associated with the need for mechanical ventilation (adjusted RR = 1.45; 95% CI: 1.04, 1.85), the Glasgow Coma Scale score < 8 (adjusted RR, 3.52; 95% CI: 2.90, 4.05), presence of comorbidity (adjusted RR, 1.47; 95% CI: 1.09, 1.83), length of stay in ICU < 24 h (adjusted RR, 1.84; 95% CI: 1.37, 2.04), oxygen saturation level, and Feeding, Analgesia, Sedation, Thrombosis prophylaxis, Head elevation, Ulcer prophylaxis, and Glucose control (FASTHUG) treatment received were significantly associated with mortality.

**Conclusion:**

The study found a high incidence proportion of death. The need for mechanical ventilation, length of stay, comorbidity, and the Glasgow Coma Scale score were significantly associated with mortality. Therefore, close monitoring and evaluation of patients are essential to improve treatment outcomes.

## Introduction

An intensive care unit (ICU) is a special hospital or healthcare facility department that provides intensive care treatment to patients with severe or life-threatening illnesses and injuries, which require constant care and close supervision under life support equipment and medication. Intensive care is the specialized treatment given to acutely unwell patients requiring critical medical care (1, 2). By 1953, Bjorn Ibsen, the anesthetist who proposed that positive pressure ventilation be the treatment of choice during the polio epidemic, had constructed Europe’s first intensive care unit; many consider him to be the “father” of intensive care (3).

Based on equipment availability and skilled medical and nursing personnel, the World Federation of Societies of Intensive Care Medicine (WFSICCM) classified ICUs into three categories. A level 1 ICU can provide oxygen and non-invasive monitoring, while a level 2 ICU can provide invasive monitoring and basic life support for a limited amount of time. A level 3 ICU offers a comprehensive range of monitoring and life support systems and may contribute to the advancement of intensive care medicine through research and education (4).

ICUs began to develop in many resource-limited countries after decades compared with the industrialized countries. However, the burden of critical illness is especially high in low-income countries as infections such as pneumonia, diarrhea, and malaria are endemic, and traffic accidents, obstetric complications, and surgical emergencies are common (5, 6).

In Ethiopia, the Ministry of Health established an Emergency Medicine and Critical Care Directorate and Implementation Guidelines in 2015 to standardize ICU organization, design, and common practices including rounds, admission, and discharge, reflecting increased attention to emergency medicine and critical care capacity (7). Currently, 53 public hospitals in Ethiopia, provide adult intensive care services (8).

Mortality is a major endpoint in epidemiological and interventional studies conducted in the ICU. Despite medical advances in patient management, ICU mortality remains high as approximately one-third of patients dying in the hospital died in ICU, though the mortality has large variations according to patient case mix and organization of care in ICU (9, 10).

Critically ill patients are admitted to the ICU to reduce morbidity and mortality associated with acute illness, trauma, or surgical procedures (11). Globally, the incidence of death in ICU roughly ranges from 20 to 47% though this magnitude varies between regions and countries (12, 13). It varies across the world depending on the ICU infrastructure, staff availability, training, pattern, and cause of ICU admission.

The incidence of mortality is high in African countries. A retrospective study conducted in Ugandan Mulago Hospital and Kenyan Hospital showed an incidence proportion of mortality of 43.7% (14) and 53.6% (15), respectively. In Ethiopia, studies have indicated poor outcomes for patients admitted to intensive care units with the mortality ranging from 39 to 47% (16–18). ICUs in Addis Ababa faced a high incidence of mortality, 32% of admitted patients to emergency care died (19), and it was 27% in Ayder Hospital (20) and 38.7% in Gondar Hospital (21).

The major causes of ICU admission include trauma, cardiac disease, acute abdominal presentations, septic shock, tetanus, and hysterectomy secondary to uterine rupture. Medical diagnoses accounted for 50.1% of ICU admissions followed by surgery (43.2%) and obstetrics (5.8%) and the corresponding survival rates of the cases were 53.6% for medical admissions, 48.0% for surgery, and 42.9% for obstetrics (22). A shortage of ICU beds, lack of sufficiently trained healthcare professionals, constraints in critical technologies, shortage of medications, scarce data on patient outcomes, immaturity of the program, lack of partnership, and stakeholders are the major challenges of critical care service in developing nations (11, 23).

Morbidity and mortality in the ICU due to critical illness or accident can have a huge physical, social, economic, and emotional impact on the life and family of the admitted patients (24). A study conducted in Malaysia showed that patients who spent a lot of money on treatment had a 2-fold increased risk of dying (25, 26). The influence of death on overall ICU expenses was studied in the United States, and the median observed cost of a unit stay was $9,619 (mean = $16,353). A national study conducted in Scotland demonstrated that ICU survivorship is associated with higher ($25,608 versus $16,913/patient) hospital resource utilization than hospital controls (27). Financial and psychological burdens, shattered family expectations and family integrity, lack of confidence in hospitals’ service delivery system, families being immersed in an unfriendly environment, and a sense of fulfillment in helping the patient were among the problems faced in ICUs by patient caregivers, according to a study conducted in Addis Ababa, Ethiopia (28).

The Ethiopian Federal Ministry of Health has shown a renewed commitment to emergency care systems through the development of the Emergency and Critical Care Directorate (ECCD) in its administrative structure. This directorate aimed to reduce unfavorable outcomes in ICUs to less than 25% by 2020 by training emergency physicians and developing out-of-hospital emergency care (7), but the incidence of mortality rate of patients admitted to ICU is still high and far beyond the target.

Despite the incidence of mortality, which is high among patients admitted to the ICU and results in loss of human and economic resources, prior studies only assessed factors associated with sociodemographic and clinical characteristics and did not assess factors associated with care in the ICU. Thus, this study aimed to fill these gaps by assessing the factors associated with mortality among patients admitted to the ICU in Nekemte City public hospitals using a retrospective cohort study method by incorporating factors related to care components.

## Methods

### Study area

The study was conducted in Western Ethiopian public hospitals on patients admitted to the adult ICU from 1 January 2021 to 31 December 2021. Nekemte Specialized Hospital and Wollega University Referral Hospitals are the two hospitals with adult ICUs in the western part of Ethiopia. The data were collected from 11 April 2022 to 11 May 2022. Wollega University Referral Hospital was established in 2017. It serves a catchment population of 3.5 million people. The ICU service provision started in 2018 and was equipped with six beds, four mechanical ventilators, one defibrillator, and one ultrasound machine. One consultant anesthesiologist, two emergency and critical care nurses, six rotating general practitioners, and ten rotating BSc nurses. Nekemte Specialized Hospital serves as a referral center for western Ethiopia, with approximately 11 million people, and ICU service provision was started recently in March 2020 GC. The ICU of the Nekemte Specialized Hospital is equipped with six beds, four mechanical ventilators, two defibrillators, and one ultrasound machine. It was also staffed by one general practitioner, one emergency and critical care nurse, one consultant physiotherapist, and nine rotating BSc nurses.

### Study design

An institutional-based retrospective cohort study design was conducted.

#### Study period

A study was conducted on data of patients admitted to adult ICU at Nekemte Specialized Hospital and Wollega University Referral Hospital from 1 January 2021 to 31 December 2021.

#### Source population

All patients who were admitted to adult intensive care units at Nekemte City public hospitals.

#### Study population

Patients who were admitted and registered into the adult ICU at Nekemte Specialized Hospital and Wollega University Referral Hospital from 1 January 2021 to 31 December 2021.

#### Inclusion criteria

All patients admitted and registered to the adult ICU of Nekemte Specialized Hospital and Wollega University Referral Hospital from 1 January 2021 to 31 December 2021 were included in the study.

#### Exclusion criteria

Patients whose charts were incomplete, patients whose charts were not found or lost, and patients referred to other higher facilities for better diagnosis and management were excluded from the study.

### Sample size determination

The sample size was calculated by using a two or double population proportion formula, and it was calculated through Epi Info version 7 statistical software package with the assumption of confidence level 95% (Z𝛼/2 = 1.96), power 80% (Z1-𝛽= 0.84), the ratio of unexposed to exposed is 2, and the proportion of outcome in exposed and RR. Independent predictors, such as the presence of sepsis, need for mechanical ventilation, hypoxia, shorter duration of ICU stay, and age greater than 80 years, were considered for sample size calculation. Among these predictors, the primary exposure variable, which was the need for mechanical ventilation, yielded the largest sample size of 414. Therefore, the final sample size of the study was 414.

### Sampling procedure

**Figure 1:**
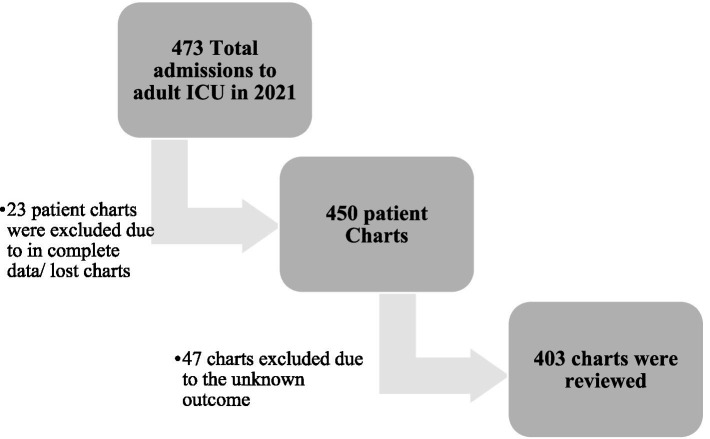
Sampling procedure of patient charts, incidence and predictors of mortality among patients admitted to adult ICU, Western Ethiopia, 2021.

The source population during the specified study period was 473, which is relatively small when compared to the minimum required sample size of 414. Therefore, a census of all patients who were registered and admitted to both ICUs was taken and analyzed. Consequently, 403 patient charts with complete data were included in the analysis (Figure 1).

### Data collection tool and procedure

A data extraction checklist was prepared after an extensive literature review and patient charts. It is comprised of three parts: sociodemographic characteristics, related clinical characteristics, and laboratory parameters. Four nurses (two for each hospital) gathered the data under the supervision of two additional BSc Nurses (one for each hospital). The medical registration number of admitted patients from the ICU registry was used to extract patient charts from the chart room. The data collectors completed the checklist by reviewing patient records and charts. Then, the supervisor checked sample data for error and completeness of the data and submitted it to the principal investigator.

### Study variables

Dependent variable: status at discharge from ICU (alive or deceased).

Independent variables: the explanatory variables were sociodemographic factors and laboratory-related and clinical factors.

### Operational definition

Deceased: those who passed away while in the ICU and given code number 3 on the ICU registry.

Alive: those who survived during ICU stay, including patients who improved and were discharged, and those who left against medical advice (code 1) and were transferred to (code 2).

FASTHUG score: FASTHUG is a shorthand representation for Feeding, Analgesia, Sedation, Thrombosis prophylaxis, Head elevation, Ulcer prophylaxis, and Glucose control. From the patient chart, whether the patient received this routine care was checked and coded ‘Yes’ if the patient had received that specific treatment, and care, and ‘No’ if that patient had not received that treatment and care. Finally, all the answers ‘yes’ were added, and the mean was calculated and yielded 3. Patients were categorized based on their scores as above or below the mean.

Comorbidity: the condition of having two or more diseases at the same time in addition to the main disease with which the patient was admitted to the ICU.

Incomplete data: patient charts with dependent variables were not found, and those with <80% of the variables were found.

### Data quality control

A one-day training was given to data collectors and supervisors on the objective of the study, the contents of the checklist, ethical issues, and the data collection approach. To identify the reliability of the data collection instruments, a pre-test was conducted before actual data collection, followed by a discussion with data collectors and supervisors, which was modified to enhance the completeness of the data. The supervisors checked the completeness of the checklist at the end of each data collection day.

### Data processing and analysis

Data were coded and entered into Epi Data Manager version 4.6.06 and exported to SPSS version 24 for cleaning and analysis. Descriptive statistics, such as frequencies, proportions, and mean with SD and median with IQR, were used to describe the data. Data were organized using tables, graphs, and charts. A binary logistic regression model was used to determine the association between each independent variable and the patient outcome at ICU discharge (29, 30). Variables with a *p*-value less than 0.25 in bivariable logistic regression were selected for multivariable logistic regression. In the multivariable model, a statistical significance was set at *p* < 0.05. The goodness of fit of the model was checked by the Hosmer–Lemeshow test with a *p*-value of 0.35, indicating that the model was well fitted, and multicollinearity was checked by VIF; values were below 10. The adjusted odds ratio (AOR) was converted to adjusted relative risk using the Zhang and Yu formula (31), based on the STROBE recommendation (32). Finally, the adjusted relative risk with 95% CI was reported.

### Ethics statement

Ethical approval was obtained from the Ethical Clearance Committee of Wollega University in its review held on 15 March 2022 (reference number WU/RD/555/2014). A letter of cooperation was written to the Nekemte Specialized Hospital and Wollega University Referral Hospital. A formal permission letter was written to the Emergency and Critical Care Department and Chart Room Office by the CED of both hospitals, and data were extracted from the patient charts. The need for informed consent was waived by the ethical review committee of Wollega University by the Declaration of Helsinki. In addition, the data collection tool was fully anonymized as it did not contain individual patient identifiers, such as name and Medical Record Numbers, and the authors had no access to the patient identifier.

## Result

### Socio-demographic characteristics of patients

A total of 403 patients were included in the analysis. The remaining 70 patient charts were excluded because of unknown patient outcomes, lost charts, or incomplete data. The mean (±SD) age of the patients admitted to adult ICU was 41 (17) years. The age category greater than 55 years constituted more admissions (28.3%), and the mortality rate was relatively high (45.2%) among the age category of 35–44 years. Of the total study participants, 221 (54.8%) were men, and 240 (59.6%) were from rural residents (Table 1).

**Table 1:** Socio-demographic characteristics of patients admitted to adult ICU, Western Ethiopia, 2021.

Characteristics category		Status		Total	
		Deceased *N* = 165	Alive *N* = 238	Count *N* = 403	%
		Count	Count		
Age category	15–24	32	48	80	20
	25–34	30	51	81	20
	35–44	28	34	62	15
	45–54	28	38	66	16
	> = 55	47	67	114	28
Sex	Male	96	125	221	55
	Female	69	113	182	45
Residence	Rural	94	146	240	60
	Urban	71	92	163	40

### Clinical characteristics of the patients

#### Admission diagnosis and disease category

Cardiovascular diseases were the most common underlying cause of ICU admission accounting for 110 (27.3%) followed by diseases of the respiratory system which contributed 79 (19.6%). The death rate was high among patients admitted with endocrine, nutritional, and metabolic diseases, of which 17 (61%) died, followed by infectious and parasitic diseases, in which 35 (54%) died (Table 2).

**Table 2:** Underlying causes of admission to adult IC, Western Ethiopia, 2021.

ESV-ICD-11 chapters	Patient status at discharge from ICU			
	Alive		Deceased	
	Count	%	Count	%
Infectious and parasitic diseases	30	46.2%	35	53.8%
Neoplasms	7	77.8%	2	22.2%
Endocrine, nutritional, or metabolic diseases	11	39.3%	17	60.7%
Diseases of the nervous system	10	76.9%	3	23.1%
Diseases of the circulatory system	75	68.2%	35	31.8%
Diseases of the respiratory system	50	63.3%	29	36.7%
Diseases of the digestive system	16	57.1%	12	42.9%
Pregnancy, childbirth, or the puerperium	9	52.9%	8	47.1%
Injury, poisoning, or certain other consequences of external causes	30	55.6%	24	44.4%
Total	238	59.1%	165	40.9%

#### Comorbidity status, source of admission, and other clinical characteristics

The study also showed that 149 (37%) patients had at least one comorbid disease, with hypertension being the most common (59, 15%), followed by diabetes mellitus (all types 19; 4%), acute and chronic kidney disease (18, 4.5%), and pneumonia (17, 4.2%). Among all admissions, 327 (81.1%) were for medical reasons, and the mortality rate was 41% in this group.

This study revealed that the median length of stay in the ICU was 72 h (IQR = 96 h). Of the patients, 285 (70.7%) stayed longer than 24 h, and 118 (29.3%) stayed for less than 24 h in ICU. Of patients who stayed less than 24 h in the ICU, 74 (62.7%) and 91 (31.9%) of patients who stayed for longer than 24 h died in ICU.

One hundred fifty-seven (39%) patients were referred from other health facilities (had referral forms), while others were self-referrals. Regarding the time of admission to the ICU, 297 (74%) patients were admitted during regular working days and the remaining 106 (26%) were admitted during weekends and holidays.

#### Vital signs and care-related characteristics

At admission, 195 (48.4%) patients had a normal respiratory rate of 12–20 breaths per minute, whereas 208 (51.6%) patients had unstable respiration of less than 12 or greater than 20 breaths per minute. The incidence proportion of mortality rates was 83 (42.6%) and 82 (39.4%) in patients with normal and deteriorated respiratory rates, respectively. Blood pressure measurement was another vital sign measured at admission to ICU; 110 (27.3%) patients were normotensive (90–140 mmHg), while 293 (72.7%) patients were either hypotensive or hypertensive. One hundred and one patients (25%) required mechanical ventilation on admission to the ICU, of whom 59 (58.4%) died, and 106 (35.1%) died among those who were not on mechanical ventilation (Table 3).

**Table 3:** Vital signs and related parameters among patients admitted to adult ICU, Western Ethiopia, 2021.

Characteristics	Patient status at discharge from ICU						
	Deceased			Alive		Total	
	Category	Count	%	Count	%	Count	%
Mechanical ventilation?	Yes	59	58.4	42	41.6	101	25.1
	No	106	35.1	196	64.9	302	74.9
Oxygen saturation	<90	101	49.3	104	50.7	205	50.9
	> = 90	64	32.3	134	67.7	198	49.1
Sepsis	Yes	39	53.4	34	46.6	73	18.1
	No	126	38.2	204	61.8	330	81.9
Glasgow Coma Scale score	< 8	122	68.2	57	31.8	179	44.4
	9–12	23	21.3	85	78.7	108	26.8
	13–15	20	17.2	96	82.8	116	28.8
BP measurement at admission	Hypotensive	40	55.6	32	44.4	72	17.9
	Normal	47	42.7	63	57.3	110	27.3
	Hypertensive	78	35.3	143	64.7	221	54.8
Respiratory rate at admission per minute	12–20	83	42.6	112	57.4	195	48.4
	< 12	4	66.7	2	33.3	6	1.5
	> 20	78	38.6	124	61.4	202	50.1
Pulse rate category	< 60	7	50.0	7	50.0	14	3.5
	60–100	101	39.8	153	60.2	254	63.0
	> 100	57	42.2	78	57.8	135	33.5
Hemoglobin count in g/dl	< 13	111	40.5	163	59.5	274	68.0
	> 13	54	41.9	75	58.1	129	32.0
FASTHUG category	0–3	59	62.8	35	37.2	94	23.3
	4–7	106	34.3	203	65.7	309	76.7

**Figure 2:**
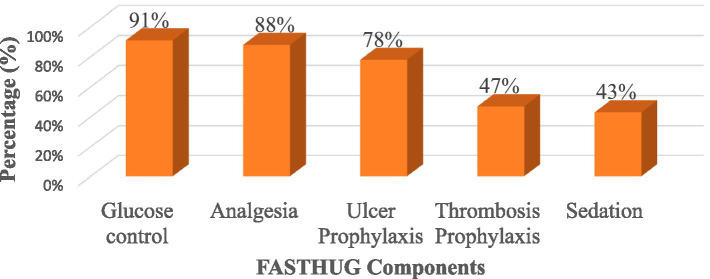
Frequency of FASTHUG treatment components among patients admitted to adult ICU; Western Ethiopia, 2021.

This study showed that 396 (98.3%) patients had received at least one component of FASTHUG treatment, and 52 (12.9%) patients received all seven components. Glucose control was the most treatment provided 367 (91.1%) followed by anti-pain provision 356 (88.3%) and ulcer prophylaxis 315 (78.2%) (Figures 1, 2).

#### Incidence and predictors of mortality in ICU

A total of 403 patients were retrospectively followed up until discharge from ICU. The incidence proportion of mortality in ICU was 40.9% (95% CI: 36.1–45.9%) in 1 year.

Eighteen variables were tested for their association with mortality at ICU discharge. In the bivariable analysis, variables such as the need for mechanical ventilation, comorbidity, length of stay in ICU, GCS, sepsis, deteriorated BP, oxygen saturation level, and FASTHUG score were significant at a *p-*value <0.25 and were selected for multivariable logistic regression at *p* value <0.25 (Table 5).

**Table 5:** Bivariable logistic regression analysis of predictors of mortality among patients admitted to adult ICU, Western Ethiopia, 2021.

Variables	Category	Status			
		Deceased	Alive	*p* Value	COR with 95%CI
Age	15–24	48	32	0.901	1
	25–34	51	30	0.864	0.95 (0.53, 1.70)
	35–44	34	28	0.555	0.84 (0.47, 1.51)
	45–54	38	28	0.614	1.17 (0.63, 2.19)
	>=55	67	47	0.875	1.05 (0.57, 1.94)
Sex	Male	125	96	1.000	1
	Female	113	69	0.262	1.28 (0.84, 1.88)
Residence	Rural	146	94	0.379	1
	Urban	92	71		1.19 (0.80, 1.80)
HIV status	Negative	219	146	0.467	1
	Positive	6	7	0.323	1.75 (0.57, 5.31)
	Unknown	13	12	0.432	1.38 (0.62, 3.12)
Mechanical ventilation	Yes	42	59	< 0.001*	2.60 (1.64, 4.12)
	No	196	106	1	1
LOS	< 24 hours	74	44	1.96 (1.62, 2.27)	1.84 (1.37−2.04)***
	> 24 hours	91	194	1	1
GCS	<= 8	57	122	0.001*	9.01 (5.70, 14.24)
	> 9	181	43	1	1
Comorbidity	Yes	75	74	0.001*	1.77 (1.17, 2.67)
	No	163	91	1	1
Pulse rate	< 60	7	7	1	1
	60–100	153	101	0.577	1.37 (0.46, 4.12)
	> 100	78	57	0.638	0.90 (0.59, 1.38)
Systolic BP	< 90	32	40	.010	1
	90–140	63	47	0.003*	2.29 (1.34, 3.94)
	> 140	143	78	0.189	1.37 (0.86, 2.18)
Respiratory rate per minute	< 12	2	4	0.339	1
	> 20	124	78	0.258	2.70 (0.48, 15.01)
	12–20	112	83	0.423	0.85 (0.57, 1.27)
Peripheral oxygen saturation	< 90	104	101	0.001*	2.03 (1.36, 3.05)
	>= 90	134	64	1.000	1
Sepsis	Yes	34	39	0.018*	1.86 (1.11, 3.10)
	No	204	126		1
The service unit from which the patient was admitted to ICU	Medical Ward	95	68	0.597	1
	Surgical Ward	26	15	0.855	0.96 (0.63, 1.48)
	Gyne Obs Ward	15	6	0.475	0.77 (0.38, 1.56)
	Emergency OPD	102	76	0.219	0.54 (0.20, 1.45)
Frequency of admission	New	232	162	0.640	1.40 (0.34, 5.67)
	Repeat	6	3		1
Anemia	Yes	75	54	0.797	1.60 (0.69, 1.62)
	No	163	111		1
FASTHUG components	<=3	35	59	0.001*	3.23 (2.00, 5.22)
	> 3	203	106		1
Time of admission to ICU	Working days	178	119		1
	Weekend and Holidays	60	46	0.550	1.15 (0.73, 1.80)

*Variables significant at *p* value <0.25; The symbol ** indicates the the *p* value of < 0.001; 1-Reference. The bold values indicate variables with *p* value less than 0.25 and candidates for multivariable analysis.

However, in the multivariable analysis, only the need for mechanical ventilation, presence of comorbidity, length of stay in the ICU, GCS, oxygen saturation level, and FASTHUG score remained statistically significant. The prediction ability of the model was checked using the area under the receiver operating characteristic curve. The area under receiver operating characteristics value was 8.853 (95% CI: 0.853, 0.889).

Patients who were on mechanical ventilation were 1.45 times more likely to die than those who were not on mechanical support (adjusted RR = 1.45, 95% CI: 1.04, 1.85). The length of stay in the ICU was another strong predictor of ICU mortality. This study showed that patients who stayed for less than 24 h were 1.84 times more likely to die than patients who stayed for longer than 24 h (adjusted RR = 1.84, 95% CI: 1.37, 2.04). This study showed that there is an inverse association between the GCS score and the risk of death in patients admitted to the ICU. Patients with a GCS of <8 were 3.52 times at risk of death in the ICU compared to those with a score greater than or equal to 9 GCS score (adjusted RR = 3.52, 95% CI: 2.90, 4.05).

Patients whose oxygen saturation of less than 90% by pulse oximetry were 1.4 times more likely to die than those with oxygen saturation greater than 90% (adjusted RR = 1.40, 95% CI: 1.03, 1.79).

Patients receiving less or equal to three components of the FASTHUG care are found to be 1.6 times at higher risk of death than those receiving four or more care components (adjusted RR, 1.60; 95% CI: 1.16, 2.01). Patients with one or more comorbidities had 1.47 times more chance of dying (Table 4).

**Table 4:** Multivariable logistic regression analysis of predictors of mortality among patients admitted to adult ICU, Western Ethiopia, 2021.

Characteristics	Category	Status		Crude RR	Adjusted RR
				(95% CI)	(95% CI)
		Deceased	Alive		
		165	238		
Mechanical ventilation	Yes	59	42	1.66 (1.34, 1.97)	1.45 (1.04–1.85)*
	No	106	196	1	1
Comorbidity	Yes	74	75	1.39 (1.10, 1.67)	1.47 (1.09–1.83)**
	No	91	163	1	1
Peripheral Oxygen saturation	< 90%	101	104	1.35 (1.15, 1.52)	1.40 (1.03–1.79)*
	> = 90%	64	134	1	1
Sepsis	Yes	39	34	1.40 (1.07, 1.72)	1.38 (0.95–1.79)
	No	126	204	1	1
Length of stay in hours	< 24 h	74	44	1.96 (1.62, 2.27)	1.84 (1.37–2.04)***
	>24 h	91	194	1	1
BP in mmHg	Normal	47	63	1	1
	Hypotensive	40	32	1.48 (1.17, 1.75)	1.36 (0.97–1.71)
	Hypertension	78	143	1.18 (0.91, 1.45)	1.05 (0.71–1.42)
GCS Score	< = 8	122	57	3.55 (3.00, 4.02)	3.52 (2.90–4.05)***
	> 9	43	181	1	1
FASTHUG care score	0–3	59	35	1.83 (1.49, 2.13)	1.60 (1.16–2.01)**
	4–7	106	203	1	1

***p-value < 0.00; **p-value < 0.01; *p-value < 0.05; 1, reference; BP, blood pressure; GCS, Glasgow Coma Scale.

## Discussion

The incidence proportion of mortality in this study was found to be comparable with the results of a study conducted in Hosanna, Gondar University, St. Paul’s Hospital, and Mulago Hospital of Uganda, which reported a mortality incidence proportion of 46.2% (16), 38.7% (21), 39% (18), and 43.7% (14), respectively. However, it was found to be lower than the result of a study conducted in Southern Ethiopia hospitals and Kenya, which reported a mortality rate of 46.8% (17) and 53.6% (15), respectively. This discrepancy might be due to the difference in admission diagnosis in which 54–80% of patients admitted in Southern Ethiopia and Western Kenyan hospitals presented with acute respiratory distress syndrome requiring respiratory support compared to the 25% in this study. This is not surprising that acute respiratory failure and acute respiratory distress syndrome are drivers of high mortality (33). The difference in the level of ICU care provided, which can be explained by differences in ICU equipment, healthcare professionals, and availability of medications could also contribute to the observed discrepancy in mortality rates between hospitals.

It is not a surprise that the incidence proportion of mortality in the current study is much higher than the finding of the study conducted in resource-affluent countries. The study conducted in Brazil reported 21% (34), and a multicenter European cohort study reported 19.1% (35). This huge discrepancy might be due to the difference in the level of ICU care, availability of medical supplies, availability of trained staff, and the use of high-cost technologies.

In this study, patients who required mechanical ventilation were more likely to die than those who were not. This finding is in line with the findings of a study conducted in Gondar (21), Kenya (15), and Brazil (34). Patients with respiratory distress are more unstable and susceptible to ventilator-associated pneumonia and other nosocomial infections, posing a risk to their clinical prognosis (36).

The Glasgow Coma Scale score was another predictor of mortality for patients in the ICU. Patients with a GCS score of <8 were more likely to die than patients with a GCS score of > = 9. This finding is similar to the finding of a study conducted in Southern Ethiopia (17), Gondar (21), and Mulago Hospital of Uganda (14). This can be explained by the fact that GCS is a significant indicator of disease severity and individuals who have suffered a head injury or other traumas are likely to have lower GCS scores (37).

Oxygen saturation level below 90% is also a predictor of death among ICU patients. This finding is similar to the finding of a study conducted in Peru and France (38, 39). Hypoxemia leads to respiratory distress syndrome due to low hemoglobin concentration in the blood. Patients may have to wait longer for ICU admission due to resource limitations and delayed hospital arrival (40).

This study also revealed that the likelihood of death decreases as the length of hospital stay increases. This finding is in agreement with a study conducted in different parts of the world (41–43). The probable cause of these early fatalities is most likely due to a late presentation to care after potential complications are developed and delayed referral from another health facility. In contrast to this study, a prospective investigation done in Turkey failed to discover a link between the length of time spent in the intensive care unit and the risk of death (42). This is due to the Turkish study’s exclusion of patients who spent less than 48 h in an intensive care unit, a period associated with a higher risk of mortality in this study.

This study found that patients who have at least one comorbidity were more likely to die than those who did not have comorbidity. This finding is consistent with the study done in West Scotland the UK (44). This can be explained by the fact that patients with comorbidities show higher Charlson Comorbidity Index and are older age patients, which are, in turn, statistically associated with mortality in the ICU (45).

FASTHUG-based care enables critically ill patients to receive holistic care that encompasses all critical issues and is proven effective in preventing adverse outcomes. This study showed that patients who had received less than three of the seven FASTHUG care components were more likely to die than those who had received at least four components of care. A comparative study conducted in Mexico and the United States revealed that the application of the FASTHUG bundle of care decreased mortality rate (46) and ventilator-associated pneumonia (47), respectively. This suggests that the nursing care model developed is effective in increasing the quality of nursing care given to critically ill patients.

### Limitations of the study

Due to the retrospective nature of the study, a limited number of variables were found; as a result, some variables like severity scores (such as APACHE II and SOFA scores) of the patients could not be found, which might have a significant association with mortality of patients admitted to ICU. This mortality incidence is not disease-specific and includes a variety of disease entities that may each have a variable mortality rate. In addition to this, factors associated with ICU resources, staffing, and training status of the staff were not assessed by this study as these factors might have an impact on the outcome of the patients.

The study followed the patients only until their discharge from the ICU and considered patients who were transferred to wards and those left against medical advice as if they were survived or alive. These patients may die after discharge, which may underestimate the incidence of mortality.

## Conclusion and recommendations

This study found a high incidence of mortality among patients admitted to the adult ICU. The severity of the condition, which is determined by the GCS score, need for mechanical ventilation, oxygen saturation level, and comorbidity significantly predict mortality among patients admitted to ICU. Patients who require mechanical ventilation were at higher risk of death because these patients were at risk of developing ventilator-associated pneumonia and the limited availability of mechanical ventilators. In addition, it can be due to poor quality of care, which is observed in the number of FASTHUG care. Hospitals should use severity scores (such as APACHE, SAPS II, and SOFA) as a crucial component at the ICU for improving clinical decisions and identifying patients with higher unexpected outcomes. Healthcare providers should also prioritize and give due attention to acutely ill patients like those with respiratory failure, lower GCS scores, and comorbidities. The application of standardized treatment protocols such as the FASTHUG bundle of care should also encouraged and applied regularly as it improves the quality of care and the favorable clinical outcomes. Future investigators should conduct a prospective follow-up study by including important variables such as severity scores and variables related to human resources, equipment, and quality of care.

## Data Availability

The original contributions presented in the study are included in the article/supplementary material, further inquiries can be directed to the corresponding author.
